# Serum Levels of Acylcarnitines Are Altered in Prediabetic Conditions

**DOI:** 10.1371/journal.pone.0082459

**Published:** 2013-12-16

**Authors:** Manuel Mai, Anke Tönjes, Peter Kovacs, Michael Stumvoll, Georg Martin Fiedler, Alexander Benedikt Leichtle

**Affiliations:** 1 Division of Endocrinology, Department for Internal Medicine, Neurology and Dermatology, University of Leipzig, Leipzig, Germany; 2 IFB Adiposity Diseases, University of Leipzig, Leipzig, Germany; 3 Center of Laboratory Medicine, University Institute of Clinical Chemistry, Inselspital – Bern University Hospital, Bern, Switzerland; Baylor College of Medicine, United States of America

## Abstract

**Objective:**

The role of mitochondrial function in the complex pathogenesis of type 2 diabetes is not yet completely understood. Therefore, the aim of this study was to investigate serum concentrations of short-, medium- and long-chain acylcarnitines as markers of mitochondrial function in volunteers with normal, impaired or diabetic glucose control.

**Methods:**

Based on a 75 g oral glucose tolerance test, 1019 studied subjects were divided into a group with normal glucose tolerance (NGT; n = 636), isolated impaired fasting glycaemia (IFG; n = 184), impaired glucose tolerance (IGT; n = 87) or type 2 diabetes (T2D; n = 112). Serum concentrations of free carnitine and 24 acylcarnitines were measured by mass spectrometry.

**Results:**

Serum levels of acetylcarnitine (C2), propionylcarnitine (C3), octanoylcarnitine (C8), malonylcarnitine/hydroxybutyrylcarnitine (C3DC+C4OH), hexanoylcarnitine (C6), octenoylcarnitine (C8:1), decanoylcarnitine (C10), decenoylcarnitine (C10:1), dodecanoylcarnitine (C12), tetradecenoylcarnitine (C14:1), tetradecadienylcarnitine (C14:2), hydroxytetradecanoylcarnitine (C14OH), hydroxyhexadecanoylcarnitine (C16OH) and octadecenoylcarnitine (C18:1) were significantly different among the groups (all p<0.05 adjusted for age, gender and BMI). Between the prediabetic states C14:1, C14:2 and C18:1 showed significantly higher serum concentrations in persons with IGT (p<0.05). Compared to T2D the IFG and the IGT subjects showed lower serum concentrations of malonylcarnitine/hydroxybutyrylcarnitine (C3DC+C4OH) (p<0.05).

**Conclusion:**

Alterations in serum concentrations of several acylcarnitines, in particular tetradecenoylcarnitine (C14:1), tetradecadienylcarnitine (C14:2), octadecenoylcarnitine (C18:1) and malonylcarnitine/hydroxybutyrylcarnitine (C3DC+C4OH) are associated not only with T2D but also with prediabetic states.

## Introduction

Obesity and insulin resistance are considered to be crucial predictors in the development of type 2 diabetes (T2D) [1]. Moreover, insulin resistance is tightly linked with alterations in lipid metabolism which may consequently lead to increased risk for coronary heart disease [2], [3].

The mitochondrial function seems to play a significant role in the complex pathogenesis of insulin resistance [4]–[6]. Acylcarnitines belong to the group of markers for mitochondrial function, specifically for beta oxidation of fatty acids. They are synthesized by the enzyme carnitine palmitoyltransferase 1 (CPT 1) that is responsible for the transport of fatty acids into the mitochondrial matrix [7], [8]. Incomplete fatty acid oxidation results in elevated acylcarnitine concentrations [9], which is used in newborn screening to detect metabolic disorders [10]. It has also been described that certain acylcarnitines are lower in obese women with polycystic ovary syndrome in comparison to controls with similar BMI [11].

Recent studies suggest that concentrations of various acylcarnitines are also associated with insulin resistance and T2D [12]–[15]. However, little is known about the relationship between alterations in serum levels and prediabetic states such as isolated impaired fasting glycaemia (IFG) or impaired glucose tolerance (IGT). Therefore, the aim of this study was to elucidate alterations in serum levels of short-, medium- and long-chain acylcarnitines with respect to the prediabetic states such as IFG or IGT in addition to normal glucose tolerance (NGT) and T2D.

## Materials and Methods

### Study population

All subjects are part of a sample from a population from Eastern Germany, the Sorbs [16], [17]. Based on a 75 g oral glucose tolerance test (oGTT) and according to the criteria of the American Diabetes Association [18] 1019 individuals were divided into groups of normal glucose tolerance (NGT, n = 636), isolated impared fasting glycaemia (IFG, n = 184), impaired glucose tolerance (IGT, n = 87) and type 2 diabetes (T2D, n = 112). Individuals with isolated IFG were defined by a fasting plasma glucose ≥5.6 mmol/l and ≤6.9 mmol/l. Patients with IGT were defined by a fasting plasma glucose <7.0 mmol/l and a 120-min plasma glucose ≥7.8 mmol/l and <11.1 mmol/l. T2D was defined by 120-min plasma glucose ≥11.1 mmol/l or fasting plasma glucose ≥7.0 mmol/l and individuals with NGT were defined by a fasting plasma glucose <5.6 mmol/l and a 120-min plasma glucose <7.8 mmol/l.

The study was approved by the ethics committee of the University Hospital of Leipzig and all subjects provided written informed consent before taking part in the study.

### Measurement of body fat content and glucose metabolism

Body fat percentage was measured by body impedance analysis (BIA). BIA was performed with BIA-2000-S (Data Input GmbH, Darmstadt, Germany) and evaluated with the software Nutri3 (Data Input GmbH). The oGTT was performed after an overnight fast with 75 g standardized glucose solution. All baseline blood samples were collected between 8:00 and 10:00 am.

### Mass spectrometric measurement of acylcarnitines

The metabolite quantitation of the serum samples was performed subsequently during 2011 with a routine method used for confirmation and follow-up diagnostics of inborn errors of metabolism in batches of clinical and randomly admixed study sample series. The targeted metabolite profiling was focused on the determination of free and 24 acyl carnitines and based on the methods of Vernez et al. [19]. For our accredited (ISO/IEC 17025, STS-№ 259) in-house method 30 µL of serum were diluted in 270 µL aqua puriss. and 30 µL of this solution were mixed with 100 µL methanolic standard solution NSK-B (Cambridge Isotopes, Andover, MA, USA; preparation according to the attached CIL instruction). Of this final solution, 4 µL were injected in our analytical system consisting of an API 4000 Q-Trap LC-MS/MS system (ABSciex, Zug, Switzerland) and an Agilent 1100 HPLC system (Agilent, Basel, Switzerland) consisting of a vacuum microdegaser, a quaternary pump, a thermostated autosampler and a column compartment module with a Macherey - Nagel column Nucleodur C8 Gravity (125×2 mm, 5 µm).

The separation was performed at a flow rate of 0.2 mL/min at 30°C. Solvent A was an aqueous solution of 250 mL water (Milli-Q-Reference; Millipore AG, Zug, Switzerland), 325 µL heptafluorobutyric acid (Fluka, Switzerland, for ion chromatography) and 170 mg ammonium acetate (Fluka, Switzerland, eluent additive for LC-MS). Solvent B was a methanolic solution of 250 mL methanol (Riedel-de Haen, Switzerland, LC-MS Chromasolv), 325 µL heptafluorobutyric acid and 170 mg ammonium acetate. A linear gradient program was used: 0 min, 95% A and 5% B, 2 min, 95%A and 5%B, 6 min, 10%A and 90%B, 14 min, 10%A and 90%B, 14.1 min, 5%A and 95%B, 16 min, 5%A and 95%B, 17 min, 95%A and 5%B, reequilibration 17–25 min.

Measurements and quantitation of the carnitines were performed by using two specific transitions (ESI positive ionization with multiple reaction monitoring) for each carnitine at unit resolution (Software Analyst 1.5, ABSciex, Zug, Switzerland) as well as in-house MS Excel™ macros. The acyl carnitine concentrations are reported in µmol/L. Carnitine, C2-, C3-, C4-, C5-, C8-, C14-, and C16-carnitine were quantified directly via the internal standard, the other carnitines in accordance to the isotope-labeled standards for C5-, C8-, C14-, and C16-carnitine with empirical correction factors derived from certified control materials (Chromsystems, MassCheck Amino Acids and Acylcarnitines Dried Blood Spot Controls, Germany and Recipe, ClinChek Whole Blood Control, Germany). A quality control plasma sample was prepared and analyzed with every batch of the study samples to verify the stability of the calculated concentrations during the measurements. For determination of the inter-day precision, provided as the percentages of the coefficient of variation (CV), 10 replicates of a quality control were analyzed on 10 different days. For concentrations ≥0.2 µmol/l the CV was <10% and for concentrations <0.2 µmol/L the CV was <35%.

### Statistical analyses

Data are shown as means ± standard deviation. Before statistical analysis, non-normally distributed parameters were logarithmically transformed to approximate a normal distribution.

A general linear model analysis including age, gender and BMI as covariates was performed for each acylcarnitine. Non-standardized residuals were then taken forward as a new dependent variable in the ANOVA analysis comparing all four phenotypic groups (NGT, IFG, IGT and T2D). For p-values <0.05 in the ANOVA a Tukey-HSD posthoc test was performed.

To assess the relationship between acylcarnitines and body fat or waist to hip ratio a linear regression model was performed adjusted for age and gender.

Statistical analysis was performed using IBM SPSS Statistics version 20.0.

## Results

Free carnitine, acetylcarnitine (C2), propionylcarnitine (C3), butyrylcarnitine (C4), isovalerylcarnitine (C5), hexanoylcarnitine (C6) and hexadecenoylcarnitine (C16:1) were significantly associated with increased body fat and waist to hip ratio (Table 1). In contrast octadecanoylcarnitine (C18) was associated with decreased body fat (p = 8.27×10^−5^, beta = −0.124) and WHR (p = 0.001, beta = −0.141). Serum concentration of octenoylcarnitine (C8:1) was associated with increased body fat (p = 4.78×10^−5^, beta = 0.131), while tetradecadienylcarnitine (C14:2) showed association with decreased body fat (p = 0.038, beta = −0.069). Hexadecanoylcarnitine (C16), hydroxytetradecanoylcarnitine (C14OH) and hydroxyhexadecanoylcarnitine (C16OH) showed positive correlations with WHR but not with body fat (Table 1).

**Table 1:** Association between acylcarnitines and body fat or waist to hip ratio.

Acylcarnitines	Body fat [%]		Waist to hip ratio	
	Beta	p-value	Beta	p-value
free carnitine	0.191	*3.73×10^−10^*	0.233	*3.33×10^−8^*
C2	0.076	*0.016*	0.114	*0.009*
C3	0.196	*2.89×10^−11^*	0.265	*7.54×10^−11^*
C4	0.08	*0.016*	0.141	*0.002*
C5	0.161	*1.36×10^−7^*	0.239	*1.24×10^−8^*
C8	−0.021	0.530	−0.05	0.270
C14	−0.012	0.715	0.045	0.338
C16	0.047	0.117	0.095	*0.023*
C3DC +C4OH	0.019	0.602	0.005	0.920
C4DC	−0.01	0.819	0.055	0.359
C5:1	−0.023	0.512	−0.027	0.579
C5OH	0.072	0.093	0.099	0.087
C5DC	−0.03	0.379	−0.025	0.599
C6	0.136	*3.79×10^−5^*	0.144	*0.002*
C8:1	0.131	*4.78×10^−5^*	0.077	0.084
C10	−0.045	0.180	−0.068	0.138
C10:1	−0.033	0.314	−0.029	0.518
C12	−0.064	0.057	−0.002	0.961
C14:1	−0.039	0.228	−0.073	0.103
C14:2	−0.069	*0.038*	−0.071	0.117
C14OH	0.03	0.428	0.178	*0.0005*
C16:1	0.091	*0.006*	0.137	*0.003*
C16OH	0.005	0.892	0.121	*0.017*
C18	−0.124	*8.27×10^−5^*	−0.141	*0.001*
C18:1	0.048	0.113	0.038	0.365

Associations were assessed in a linear regression model adjusted for age and gender. P-values <0.05 were considered as statistically significant and marked in bold italic.

However, there were no associations between body fat or WHR with other acylcarnitines (Table 1).

A group of 410 men and 609 women with an age range from 18 to 88 years and BMI from 15.4 to 50.3 kg/m^2^ were divided into groups of NGT, IFG, IGT and T2D (Table 2). Age, BMI and the other parameters (% body fat, waist and hip circumference, WHR) were significantly different in comparison of all groups (p<0.001).

**Table 2:** Characteristics of the study population (N = 1019).

	NGT	IFG	IGT	T2D	p-value
N (male/female)	636 (215/421)	184 (112/72)	87 (35/52)	112 (48/64)	
Age (years)	42.1±14.9	53.8±13.5	59.2±13.1	63.7±11.4	<0.001
	[18–80]	[19–88]	[20–82]	[20–87]	
BMI (kg/m^2^)	25.3±4.2	28.9±4.5	29.9±4.7	30.9±5.5	<0.001
	[15.4–47.4]	[19–46.3]	[22.2–43]	[18.8–50.3]	
Body fat (%)	19.2±8.2	22.9±8.9	26.2±9.9	26.9±10.5	<0.001
	[5.3–65.4]	[8.4–56]	[11.1–51.9]	[7.5–58.8]	
Waist circumference (cm)	85.5±11.9	98.2±11.8	99.5±11.2	102.5±13.9	<0.001
	[59–139]	[67–133]	[72–122]	[69–139]	
Hip circumference (cm)	101.6±7.8	106.1±9	108.3±10.3	108.6±10.6	<0.001
	[70–141]	[90–146]	[88–140]	[88–146]	
Waist to hip ratio	0.84±0.09	0.93±0.09	0.92±0.09	0.94±0.09	<0.001
	[0.56–1.3]	[0.71–1.13]	[0.74–1.15]	[0.65–1.18]	

± standard deviation and ranges are given for each variable. NGT, normal glucose tolerance; IFG, isolated impaired fasting glycaemia; IGT, impaired glucose tolerance; T2D, type 2 diabetes Group specific arithmetic means

In comparison of the four groups there were significant (age, gender and BMI adjusted) differences in the serum levels of acetylcarnitine (C2), propionylcarnitine (C3), octanoylcarnitine (C8), malonylcarnitine/hydroxybutyrylcarnitine (C3DC+C4OH), hexanoylcarnitine (C6), octenoylcarnitine (C8:1), decanoylcarnitine (C10), decenoylcarnitine (C10:1), dodecanoylcarnitine (C12), tetradecenoylcarnitine (C14:1), tetradecadienylcarnitine (C14:2), hydroxytetradecanoylcarnitine (C14OH), hydroxyhexadecanoylcarnitine (C16OH) and octadecenoylcarnitine (C18:1) (Table 3).

**Table 3:** Acylcarnitines serum concentration in individuals with normal glucose tolerance (NGT), isolated impaired fasting glycaemia (IFG), impaired glucose tolerance (IGT) and type 2 diabetes (T2D).

						p-value Tukey-HSD					
Acylcarnitines (µmol/l)	NGT	IFG	IGT	T2D	p-value adj. ANOVA	NGT vs IFG	NGT vs IGT	NGT vs T2D	IFG vs IGT	IFG vs T2D	IGT vs T2D
C2	8.711±3.125	10.068±3.317	11.223±3.394	12.1±4.091	*0.00075*	0.997	*0.048*	*0.003*	0.140	*0.027*	0.979
	[2.743–23.372]	[4.714–22.655]	[6.041–24.841]	[4.675–32.938]							
C3	0.451±0.155	0.574±0.167	0.551±0.178	0.632±0.24	*0.016*	0.095	0.990	0.072	0.293	0.967	0.193
	[0.146–1.165]	[0.180–1.181]	[0.235–1.018]	[0.266–2.37]							
C8	0.210±0.121	0.239±0.143	0.256±0.105	0.282±0.157	*0.032*	1.000	0.305	0.058	0.400	0.126	0.976
	[0.036–1.066]	[0.081–1.203]	[0.098–0.537]	[0.084–1.096]							
C3DC+ C4OH	0.038±0.027	0.042±0.030	0.055±0.038	0.076±0.059	*1.7x10^−6^*	0.457	0.808	*8.3x10^−6^*	0.293	*1.97x10^−6^*	*0.032*
	[0–0.130]	[0–0.138]	[0–0.252]	[0–0.492]							
C6	0.061±0.082	0.068±0.030	0.076±0.028	0.083±0.036	*0.025*	0.937	0.078	*0.007*	0.308	0.088	0.979
	[0.007–2.008]	[0.017–0.252]	[0.031–0.156]	[0.026–0.231]							
C8:1	0.161±0.096	0.202±0.128	0.246±0.138	0.277±0.163	*0.004*	0.996	0.102	*0.011*	0.245	0.062	0.980
	[0.025–0.799]	[0.051–0.951]	[0.056–0.709]	[0.058–0.717]							
C10	0.334±0.22	0.379±0.251	0.42±0.209	0.448±0.286	*0.018*	1.000	0.095	0.089	0.171	0.183	0.998
	[0.038–2.042]	[0.076–1.992]	[0.133–1.039]	[0.107–1.954]							
C10:1	0.164±0.084	0.184±0.089	0.199±0.086	0.226±0.102	*0.013*	0.692	0.773	*0.033*	0.410	*0.011*	0.636
	[0.023–0.640]	[0.044–0.585]	[0.08–0.505]	[0.044–0.833]							
C12	0.098±0.057	0.106±0.058	0.122±0.057	0.127±0.065	*0.044*	0.531	0.473	0.303	0.141	0.071	1.000
	[0–0.445]	[0–0.332]	[0.006–0.309]	[0.002–0.423]							
C14:1	0.08±0.045	0.087±0.043	0.109±0.048	0.111±0.053	*4.5x10^−5^*	0.387	*0.007*	*0.022*	*0.001*	*0.002*	0.955
	[0.005–0.412]	[0.017–0.247]	[0.044–0.299]	[0.038–0.356]							
C14:2	0.033±0.019	0.035±0.017	0.042±0.019	0.044±0.023	*0.0004*	0.211	0.067	0.067	*0.004*	*0.003*	0.997
	[0.003–0.156]	[0.002–0.091]	[0.015–0.113]	[0.013–0.159]							
C14OH	0.012±0.009	0.010±0.008	0.009±0.006	0.009±0.006	*0.032*	0.158	0.397	0.125	1.000	0.980	0.990
	[0–0.042]	[0–0.035]	[0–0.032]	[0–0.036]							
C16OH	0.004±0.003	0.004±0.003	0.004±0.003	0.005±0.003	*0.008*	0.522	1.000	*0.023*	0.808	*0.004*	0.189
	[0–0.032]	[0–0.015]	[0–0.011]	[0–0.015]							
C18:1	0.102±0.032	0.115±0.033	0.129±0.036	0.133±0.041	*0.003*	0.812	*0.039*	0.097	*0.019*	*0.048*	0.964
	[0.029–0.213]	[0.055–0.226]	[0.061–0.224]	[0.059–0.252]							
free carnitine	38.879±10.324	46.210±10.069	45.489±9.9	45.855±11.319	0.103						
	[17.354–82.377]	[17.178–80.305]	[24.61–67.65]	[24.576–87.08]							
C4	0.225±0.107	0.268±0.124	0.273±0.124	0.295±0.122	0.077						
	[0.047–1.113]	[0.068–1.121]	[0.108–0.923]	[0.104–0.886]							
C5	0.123±0.045	0.147±0.045	0.146±0.043	0.158±0.047	0.062						
	[0.045–0.428]	[0.057–0.323]	[0.058–0.246]	[0.066–0.294]							
C14	0.038±0.016	0.041±0.019	0.042±0.016	0.046±0.021	0.619						
	[0–0.113]	[0–0.123]	[0.003–0.102]	[0–0.131]							
C16	0.113±0.031	0.130±0.032	0.134±0.032	0.138±0.035	0.084						
	[0.044–0.282]	[0.067–0.249]	[0.062–0.228]	[0.073–0.228]							
C4DC	0.009±0.011	0.012±0.012	0.01±0.012	0.016±0.015	0.112						
	[0 –0.050]	[0–0.046]	[0–0.041]	[0–0.058]							
C5:1	0.012±0.007	0.012±0.007	0.012±0.006	0.014±0.008	0.396						
	[0–0.048]	[0.001–0.041]	[0.002–0.032]	[0.001–0.043]							
C5OH	0.011±0.011	0.013±0.012	0.013±0.011	0.014±0.012	0.594						
	[0–0.072]	[0–0.048]	[0–0.042]	[0–0.046]							
C5DC	0.121±0.042	0.135±0.050	0.135±0.042	0.129±0.049	0.053						
	[0.021–0.289]	[0.052–0.294]	[0.066–0.268]	[0–0.294]							
C16:1	0.021±0.01	0.024±0.012	0.028±0.014	0.029±0.013	0.196						
	[0–0.067]	[0.001–0.074]	[0.001–0.066]	[0.01–0.093]							
C18	0.059±0.02	0.067±0.019	0.064±0.019	0.068±0.02	0.770						
	[0.018–0.176]	[0.027–0.124]	[0.03–0.105]	[0.029–0.114]							

± standard deviation and ranges. P-values are corrected for age, gender and BMI. Tukey-HSD post-hoc test was performed only when the adjusted ANOVA showed significant differences. Data present means

**Figure 1:**
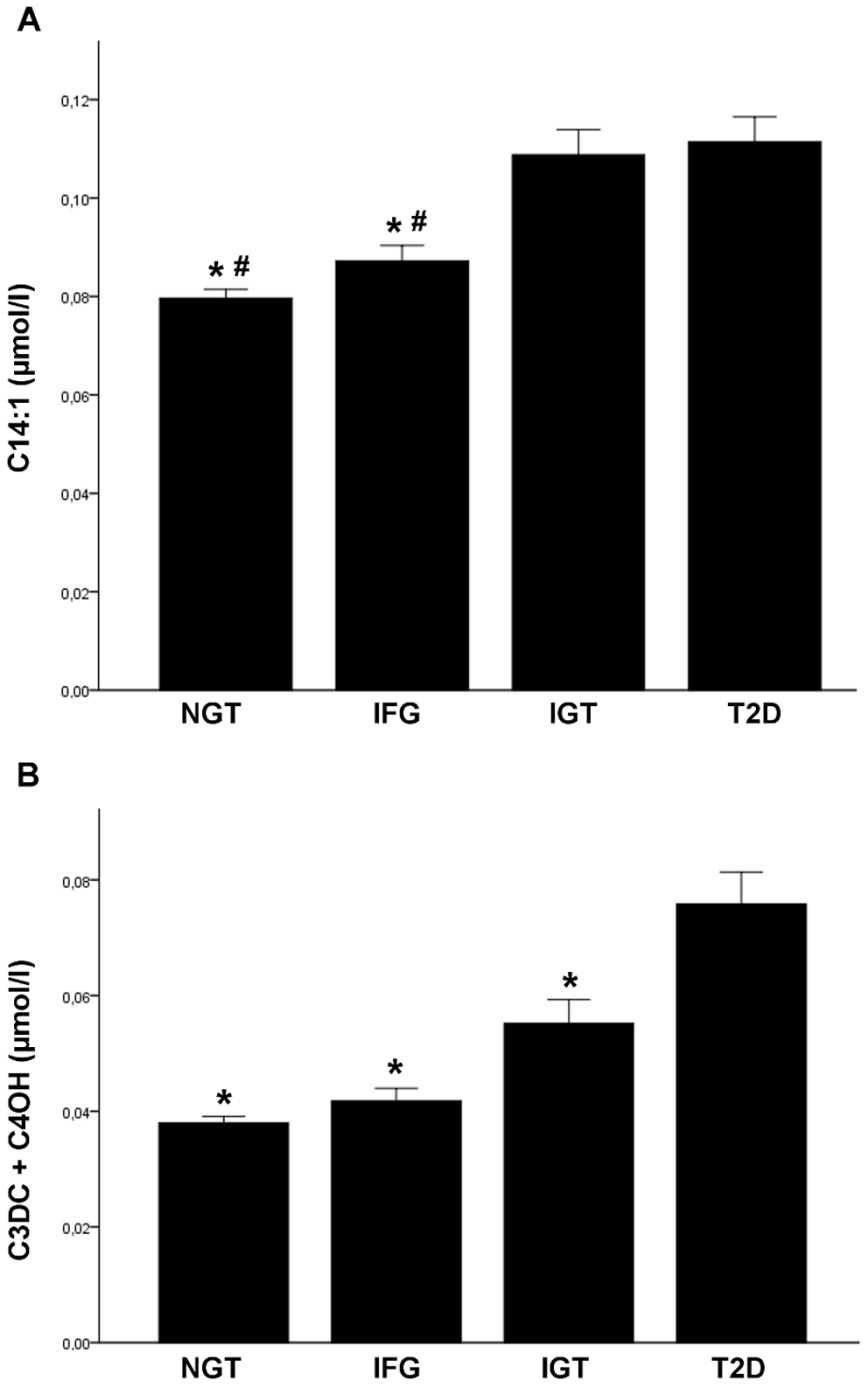
Serum concentration for C14:1-carnitine (A) and C3DC+C4OH-carnitine (B) in the different groups. Shown are means + SEM for NGT (normal glucose tolerance), IFG (impaired fasting glycaemia), IGT (impaired glucose tolerance) and T2D (type 2 diabetes); * p<0.05 vs. T2D, # p<0.01 vs. IGT

Persons with IGT had higher concentrations of C2, C14:1 and C18:1 compared to individuals with NGT, while none of the acylcarnitines showed a significant difference of serum levels in comparison of NGT and IFG. Patients with T2D showed a significantly increased concentration of C2, C3DC+C4OH, C6, C8:1, C10:1, C14:1, and C16OH in comparison of individuals with NGT. Only C14:1 (p = 0.001), C14:2 (p = 0.004) and C18:1 (p = 0.019) showed a significant altered serum level between IFG and IGT. Compared to patients with T2D, concentrations of C3DC+C4OH were significantly lower in individuals with IFG (p = 1.97×10^−6^) or IGT (p = 0.032) (Figure 1).

However, there were no significant differences in serum levels of free carnitine, butyrylcarnitine (C4), isovalerylcarnitine (C5), tetradecanoylcarnitine (C14), hexadecanoylcarnitine (C16), succinylcarnitine (C4DC), tiglylcarnitine (C5:1), hydroxyvalerylcarnitine (C5OH), glutarylcarnitine (C5DC), hexadecenoylcarnitine (C16:1) and octadecanoylcarnitine (C18) between the groups (Table 3).

## Discussion

The clinical relevance of differentiating the prediabetic states of isolated impaired fasting glycaemia and impaired glucose tolerance has previously been shown by others [20], [21]. It has been suggested that different pathomechanisms lead to either an isolated IFG or an isolated IGT [21]. However, little is known about the role of acylcarnitines in differentiating these prediabetic states from each other as well as from normal glucose tolerance or T2D. We addressed this issue in the present study. Our analyses reveal significant differences in concentrations of C2, C3, C8, C3DC+C4OH, C6, C8:1, C10, C10:1, C12, C14:1, C14:2, C14OH, C16OH and C18:1 between the prediabetic conditions. The most profound relationship with the metabolic phenotypes was observed for the serum concentration of malonylcarnitine/hydroxybutyrylcarnitine (C3DC+C4OH). C3DC+C4OH was also the only metabolite which was significantly higher in patients with T2D in comparison to individuals with IGT. On the other hand, tetradecenoylcarnitine (C14:1), tetradecadienylcarnitine (C14:2) and octadecenoylcarnitine (C18:1) were the only acylcarnitines that showed a significant difference between the two prediabetic states in our study. Others have reported that C14:1 decreased after glucose ingestion [22] and increased after a fasting period [23]. This might be due to the higher fasting free fatty acid plasma concentrations in IGT subjects when compared with persons with IFG [24]. However, it remains to be elucidated why these were the only carnitines differentially regulated in IGT and IFG.

Higher acetylcarnitine (C2) levels in IGT subjects compared to NGT have been shown previously [25]. We could confirm this association in our study.

Several acylcarnitines were significantly increased in patients with T2D in comparison with individuals with NGT. One could argue that T2D is typically associated with higher body fat percentage and higher load of free fatty acids [26], but some of the acylcarnitines, such as C3DC+C4OH, C10:1 or C14:1, were not associated with body fat in our study. It was speculated that acylcarnitines are involved in the linkage between amino-acid-metabolism and type 2 diabetes [27]. Fiehn et al. showed that concentrations of leucine and valine were increased in type 2 diabetic obese women and also correlated with concentrations of plasma acylcarnitines [27]. So maybe a diabetes specific dysregulation of fatty acid oxidation and mitochondrial function leads to the higher amount of these acylcarnitines.

In the present study we confirmed previously reported associations between acylcarnitines serum concentrations and body fat [28]. We showed, that percentage of body fat correlated positively with serum levels of free carnitine and the acylcarnitines C2, C3, C4, C5, C6, C8:1 and C16:1, but negatively with acylcarnitines C14:2 and C18. It is likely that the higher body fat correlates with an upregulated or maybe overloaded beta oxidation of fatty acids, which leads predominantly to higher amounts of short- or medium-chain-acylcarnitines.

It has been shown previously that long-chain-acylcarnitines decreased after a long aerobic exercise program in obese women [29]. In contrast, medium-chain acylcarnitines were reported to be increased after moderate intensity exercise [30]. Furthermore, an association between impaired muscle function and acylcarnitines has been described [31].

It could be shown that medium-chain acylcarnitines increase in persons with high fasting respiratory quotient as a risk factor for metabolic syndrome [32]. So these acylcarnitines are markers for incomplete fatty acid oxidation. In our study medium-chain and long-chain acylcarnitines were associated with the two prediabetic states, suggesting a linkage between the differences in prediabetic states and an incomplete or overloaded fatty acid oxidation.

Supported by our findings that certain acylcarnitines did not show any association with body fat or WHR, fat mass and fat distribution do not seem to be the only factors possibly explaining higher serum levels of these metabolites.

In conclusion, our study showed that alterations in serum concentrations of several acylcarnitines, in particular decenoylcarnitine (C14:1), tetradecadienylcarnitine (C14:2), octadecenoylcarnitine (C18:1) and malonylcarnitine/hydroxybutyrylcarnitine (C3DC+C4OH) are associated not only with T2D but also with prediabetic states. The data further support the existence of various pathomechanisms being responsible for prediabetic states of IFG or IGT. Finally, our findings suggest that higher concentrations of acylcarnitines associated with T2D cannot be explained by exclusively obesity.
